# *tert*-Butyl 2-benzoyl-2-methyl­propanoate

**DOI:** 10.1107/S1600536810003120

**Published:** 2010-01-30

**Authors:** Marshall W. Logue, Rudy L. Luck, Nicklaus S. Maynard, Sandra S. Orlowski, Louis R. Pignotti, Annie L. Putman, Kelli M. Whelan

**Affiliations:** a Department of Chemistry, 1400 Townsend Drive, Michigan Technological University, Houghton, MI 49931, USA

## Abstract

The title compound, C_15_H_20_O_3_, is bent with a dihedral angle of 67.28 (9)° between the mean planes of the phenyl ring and a group encompassing the ester functionality (O=C—O—C). In the crystal, mol­ecules related by inversion symmetry are connected by weak C—H⋯O inter­actions into infinite chains. On one side of the mol­ecule there are two adjacent inter­actions between neighbouring mol­ecules involving the H atoms of methyl groups from the dimethyl groups and the O atoms of the ketone; on the other side, there are also two inter­actions to another adjacent mol­ecule involving the H atoms on the phenyl rings and the carbonyl O atoms of the ester functionality.

## Related literature

For the synthesis, spectroscopic characterization and reactivity of the title compound, see: Logue (1974 ▶); Logue *et al.* (1975 ▶). For related structures, see: Crosse *et al.* (2010*a* ▶,*b* ▶); Gould *et al.* (2010 ▶). For the syntheses and characterization of structurally similar indanone-derived β-keto ester derivatives, see: Mouri *et al.* (2009 ▶); Noritake *et al.* (2008 ▶); Rigby & Dixon (2008 ▶). For weak hydrogen-bonded inter­actions, see: Karle *et al.* (2009 ▶).
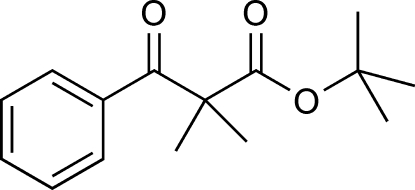


## Experimental

### 

#### Crystal data

C_15_H_20_O_3_
*M* *_r_* = 248.31
Triclinic, 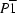

*a* = 8.616 (3) Å
*b* = 8.696 (3) Å
*c* = 11.310 (5) Å
α = 73.25 (4)°
β = 72.25 (3)°
γ = 66.05 (3)°
*V* = 724.3 (5) Å^3^
*Z* = 2
Mo *K*α radiation
μ = 0.08 mm^−1^
*T* = 291 K
0.40 × 0.35 × 0.30 mm

#### Data collection

Enraf–Nonius TurboCAD-4 diffractometer
Absorption correction: ψ scan (North *et al.*, 1968 ▶) *T*_min_ = 0.965, *T*_max_ = 0.979
2735 measured reflections
2548 independent reflections
1689 reflections with *I* > 2σ(*I*)
*R*_int_ = 0.015
3 standard reflections every 166 min  intensity decay: 1%

#### Refinement

*R*[*F* ^2^ > 2σ(*F* ^2^)] = 0.043
*wR*(*F* ^2^) = 0.119
*S* = 1.02
2548 reflections
164 parameters
H-atom parameters constrained
Δρ_max_ = 0.12 e Å^−3^
Δρ_min_ = −0.12 e Å^−3^

### 

Data collection: *CAD-4 EXPRESS* (Enraf–Nonius, 1994 ▶); cell refinement: *CAD-4 EXPRESS*; data reduction: *XCAD4* (Harms & Wocadlo, 1995 ▶); program(s) used to solve structure: *SIR2004* (Burla *et al.*, 2005 ▶); program(s) used to refine structure: *SHELXL97* (Sheldrick, 2008 ▶); molecular graphics: *ORTEP-3 for Windows* (Farrugia, 1997 ▶) and *Mercury* (Macrae *et al.*, 2008 ▶); software used to prepare material for publication: *WinGX* (Farrugia, 1999 ▶) and *publCIF* (Westrip, 2010 ▶).

## Supplementary Material

Crystal structure: contains datablocks global, I. DOI: 10.1107/S1600536810003120/zl2265sup1.cif

Structure factors: contains datablocks I. DOI: 10.1107/S1600536810003120/zl2265Isup2.hkl

Additional supplementary materials:  crystallographic information; 3D view; checkCIF report

## Figures and Tables

**Table 1:** Hydrogen-bond geometry (Å, °)

*D*—H⋯*A*	*D*—H	H⋯*A*	*D*⋯*A*	*D*—H⋯*A*
C5—H5⋯O2^i^	0.93	2.66	3.317 (3)	128
C9—H9*A*⋯O1^ii^	0.96	2.65	3.557 (3)	158

Symmetry codes: (i) 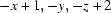; (ii) 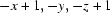.

**Fig. 1.**
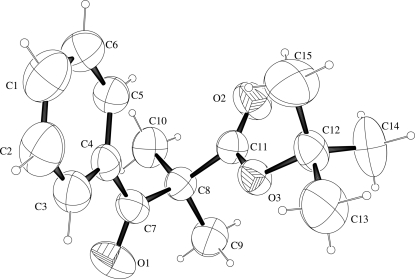
ORTEP-3 (Farrugia, 1997) drawing of the title compound with displacement ellipsoids drawn at the 50% probability level.

**Fig. 2.**
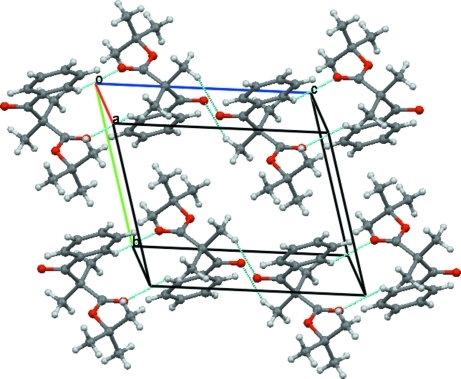
A Mercury (Macrae et al., 2008) illustration of the H-bonded linkages in the title compound using blue dashed lines showing the relationships between non-interacting chains.

## supplementary crystallographic information

### Comment

Treatment of 2,2-disubstituted *t*-butyl β-keto esters with trifluoroacetic acid at room temperature quantitatively generates the corresponding 2,2-disubstituted β-keto acids, which were used to probe the nature of the transition state for the thermal decarboxylation of β-keto acids (Logue *et al.*, 1975). Structurally similar indanone-derived β-keto ester derivatives have been prepared recently (Mouri *et al.*, 2009; Noritake *et al.*, 2008; Rigby & Dixon, 2008). The directing nature of weak C—H···O H-bonds has been noted to be of importance to afford the three dimensional structure observed in these kinds of molecules (Karle *et al.*, 2009).

In this contribution we present the solid state structure of one such 2,2-disubstituted β-keto acid, i.e. the title compound being the unsubstituted phenyl derivative. This is the first paper in a series of four dealing with substituted derivatives (H– (this paper), CH_3_–, Cl- and NO_2_– on the *para*-position of the phenyl ring) of the title compound. A more detailed comparison of all four substitution compounds will be given in the fourth paper of this series (Crosse *et al.*, 2010*a*).

The molecule, Fig. 1, displays a bent geometry with a dihedral angle between the mean planes of the phenyl ring and a plane composed of the ester functionality of 67.28 (9)°. Molecules are linked by weak C—H···O hydrogen bonds generating infinite chains parallel to the *c* axis as shown in Fig. 2. On one side of the molecule there are two adjacent interactions between neighbouring molecules involving H-atoms on methyl groups from the dimethyl moiety and O-atoms on the ketone; on the other side there are also two interactions to another adjacent molecule involving H-atoms on the phenyl rings and the carbonyl O-atoms on the ester functionality.The phenyl rings are not involved in intercalation or stacking interactions either within or between the chains. Instead neighbouring *t*-butyl groups on adjacent chains exhibit hydrophobic stacking.

### Experimental

Crystals of the material were synthesized as reported earlier and were grown by evaporation of a solution in hexane (Logue, 1974). IR (neat, cm^-1^): 3439 (br), 3077 (*m*), 2980 (*s*), 2938 (*m*), 1729 (*s*), 1683 (*s*), 1598 (*m*) 1580 (*m*), 1447 (*m*), 1388 (*m*), 1367 (*m*), 1279 (*s*), 1156 (*s*), 987 (*m*), 928 (*m*), 845 (*m*), 796 (*m*), 709 (*s*). ^1^H NMR (CDCl_3_,) δ: 1.26 (s, 9*H*), 1.49 (s, 6*H*), 7.39 (t, 2*H*, J=6.8 Hz), 7.49 (t, 1*H*, J=7.6 Hz), 7.85 (d, 2*H*, J=7.2 Hz). ^13^C NMR (CDCl_3_) δ: 24.1, 27.7, 54.3, 81.9, 128.5, 128.9, 132.7, 135.7, 174.2, 198.4.

### Refinement

All H atoms were placed at calculated positions, with C—H = 0.93 Å (aromatic) or 0.96 Å (methyl) and refined using a riding model with *U*_iso_(H) constrained to be 1.5 *U*_eq_(C) for methyl groups and 1.2 *U*_eq_(C) for all other C atoms. The quality of the data as reflected by only 66% of the reflections observed, large ADP's and inaccurate C—C bond lengths is low. The data had been collected on a 30 year old single point detector instrument not equipped with a low temperature device as part of a class project with undergraduate students. Due to the time constraints imposed by the class schedule a maximum exposure time of 60 s had to be alloted for measuring each reflection.

### Crystal data

**Table:**

C_15_H_20_O_3_	*Z* = 2
*M**_r_* = 248.31	*F*(000) = 268
Triclinic, *P*1	*D*_x_ = 1.139 Mg m^−^^3^
Hall symbol: -P 1	Mo *K*α radiation, λ = 0.71073 Å
*a* = 8.616 (3) Å	Cell parameters from 25 reflections
*b* = 8.696 (3) Å	θ = 10–15°
*c* = 11.310 (5) Å	µ = 0.08 mm^−^^1^
α = 73.25 (4)°	*T* = 291 K
β = 72.25 (3)°	Prism, colourless
γ = 66.05 (3)°	0.40 × 0.35 × 0.30 mm
*V* = 724.3 (5) Å^3^	

### Data collection

**Table:**

Enraf–Nonius TurboCAD-4 diffractometer	1689 reflections with *I* > 2σ(*I*)
Radiation source: Enraf Nonius FR590	*R*_int_ = 0.015
graphite	θ_max_ = 25.0°, θ_min_ = 1.9°
non–profiled ω/2τ scans	*h* = 0→10
Absorption correction: ψ scan (North *et al.*, 1968)	*k* = −9→10
*T*_min_ = 0.965, *T*_max_ = 0.979	*l* = −12→13
2735 measured reflections	3 standard reflections every 166 min
2548 independent reflections	intensity decay: 1%

### Refinement

**Table:**

Refinement on *F*^2^	Secondary atom site location: difference Fourier map
Least-squares matrix: full	Hydrogen site location: inferred from neighbouring sites
*R*[*F*^2^ > 2σ(*F*^2^)] = 0.043	H-atom parameters constrained
*wR*(*F*^2^) = 0.119	*w* = 1/[σ^2^(*F*_o_^2^) + (0.0496*P*)^2^ + 0.1543*P*] where *P* = (*F*_o_^2^ + 2*F*_c_^2^)/3
*S* = 1.02	(Δ/σ)_max_ < 0.001
2548 reflections	Δρ_max_ = 0.12 e Å^−^^3^
164 parameters	Δρ_min_ = −0.12 e Å^−^^3^
0 restraints	Extinction correction: *SHELXL97* (Sheldrick, 2008)
Primary atom site location: structure-invariant direct methods	Extinction coefficient: 0.064 (6)

### Special details

**Table:**

Experimental. Number of psi-scan sets used was 5. Theta correction was applied. Averaged transmission function was used. No Fourier smoothing was applied.
Geometry. All s.u.'s (except the s.u. in the dihedral angle between two l.s. planes) are estimated using the full covariance matrix. The cell s.u.'s are taken into account individually in the estimation of s.u.'s in distances, angles and torsion angles; correlations between s.u.'s in cell parameters are only used when they are defined by crystal symmetry. An approximate (isotropic) treatment of cell s.u.'s is used for estimating s.u.'s involving l.s. planes.
Refinement. Refinement of *F*^2^ against ALL reflections. The weighted *R*-factor *wR* and goodness of fit *S* are based on *F*^2^, conventional *R*-factors *R* are based on *F*, with *F* set to zero for negative *F*^2^. The threshold expression of *F*^2^ > 2σ(*F*^2^) is used only for calculating *R*-factors(gt) *etc*. and is not relevant to the choice of reflections for refinement. *R*-factors based on *F*^2^ are statistically about twice as large as those based on *F*, and *R*- factors based on ALL data will be even larger.

### Fractional atomic coordinates and isotropic or equivalent isotropic displacement parameters (Å^2^)

**Table:**

	*x*	*y*	*z*	*U*_iso_*/*U*_eq_	
C1	1.0848 (3)	−0.3377 (3)	0.8308 (3)	0.0843 (7)	
H1	1.1732	−0.4115	0.8727	0.101*	
C2	1.1205 (3)	−0.2955 (3)	0.7023 (3)	0.0828 (7)	
H2	1.2336	−0.3389	0.6568	0.099*	
C3	0.9887 (3)	−0.1880 (3)	0.6390 (2)	0.0663 (6)	
H3	1.0133	−0.1619	0.5511	0.08*	
C4	0.8198 (2)	−0.1190 (2)	0.70634 (18)	0.0499 (5)	
C5	0.7874 (3)	−0.1610 (3)	0.83672 (19)	0.0572 (5)	
H5	0.6759	−0.1144	0.8835	0.069*	
C6	0.9192 (3)	−0.2718 (3)	0.8982 (2)	0.0715 (6)	
H6	0.8953	−0.3017	0.986	0.086*	
C7	0.6830 (3)	−0.0021 (3)	0.63224 (18)	0.0535 (5)	
O1	0.7093 (2)	−0.0062 (2)	0.52144 (14)	0.0808 (5)	
C8	0.5114 (2)	0.1223 (2)	0.69448 (18)	0.0521 (5)	
C9	0.4249 (3)	0.2636 (3)	0.5920 (2)	0.0755 (7)	
H9B	0.5024	0.3236	0.5419	0.113*	
H9C	0.3191	0.3425	0.6314	0.113*	
H9A	0.3993	0.2127	0.5387	0.113*	
C10	0.3865 (3)	0.0253 (3)	0.7712 (2)	0.0707 (6)	
H10A	0.3667	−0.0282	0.7166	0.106*	
H10B	0.2779	0.1043	0.8075	0.106*	
H10C	0.4368	−0.0608	0.8375	0.106*	
C11	0.5454 (2)	0.2112 (2)	0.77688 (18)	0.0505 (5)	
O2	0.4621 (2)	0.2339 (2)	0.88045 (14)	0.0736 (5)	
O3	0.67818 (16)	0.26535 (16)	0.71377 (11)	0.0533 (4)	
C12	0.7438 (3)	0.3557 (3)	0.7690 (2)	0.0616 (6)	
C13	0.8897 (3)	0.3905 (4)	0.6630 (3)	0.0942 (9)	
H13A	0.845	0.4548	0.5885	0.141*	
H13B	0.9783	0.2838	0.6455	0.141*	
H13C	0.9384	0.4551	0.6876	0.141*	
C14	0.6027 (4)	0.5217 (3)	0.7977 (3)	0.1024 (10)	
H14A	0.5558	0.5852	0.724	0.154*	
H14B	0.6503	0.588	0.8214	0.154*	
H14C	0.512	0.4977	0.8659	0.154*	
C15	0.8130 (4)	0.2379 (4)	0.8824 (3)	0.1046 (10)	
H15A	0.8952	0.1314	0.8593	0.157*	
H15B	0.7186	0.2168	0.9482	0.157*	
H15C	0.8692	0.2902	0.9121	0.157*	

### Atomic displacement parameters (Å^2^)

**Table:**

	*U*^11^	*U*^22^	*U*^33^	*U*^12^	*U*^13^	*U*^23^
C1	0.0638 (15)	0.0716 (16)	0.103 (2)	−0.0149 (12)	−0.0227 (14)	−0.0031 (14)
C2	0.0506 (13)	0.0702 (15)	0.104 (2)	−0.0101 (12)	0.0024 (13)	−0.0182 (14)
C3	0.0647 (14)	0.0601 (13)	0.0670 (13)	−0.0244 (12)	0.0047 (11)	−0.0175 (11)
C4	0.0526 (11)	0.0448 (10)	0.0568 (11)	−0.0232 (9)	−0.0039 (9)	−0.0154 (9)
C5	0.0517 (11)	0.0579 (12)	0.0588 (12)	−0.0185 (10)	−0.0068 (10)	−0.0128 (10)
C6	0.0676 (15)	0.0702 (14)	0.0704 (14)	−0.0210 (12)	−0.0186 (12)	−0.0048 (12)
C7	0.0608 (12)	0.0618 (12)	0.0526 (11)	−0.0349 (10)	−0.0080 (9)	−0.0165 (9)
O1	0.0838 (11)	0.1121 (13)	0.0602 (10)	−0.0381 (10)	−0.0145 (8)	−0.0314 (9)
C8	0.0507 (11)	0.0547 (11)	0.0587 (11)	−0.0244 (9)	−0.0149 (9)	−0.0099 (9)
C9	0.0812 (16)	0.0708 (15)	0.0853 (16)	−0.0268 (12)	−0.0395 (13)	−0.0069 (12)
C10	0.0558 (12)	0.0752 (15)	0.0921 (16)	−0.0357 (11)	−0.0118 (11)	−0.0172 (12)
C11	0.0473 (11)	0.0503 (11)	0.0544 (11)	−0.0187 (9)	−0.0091 (9)	−0.0101 (9)
O2	0.0743 (10)	0.0925 (11)	0.0624 (9)	−0.0433 (9)	0.0091 (8)	−0.0310 (8)
O3	0.0550 (8)	0.0628 (8)	0.0547 (8)	−0.0329 (7)	−0.0061 (6)	−0.0173 (6)
C12	0.0660 (13)	0.0680 (13)	0.0706 (14)	−0.0348 (11)	−0.0167 (11)	−0.0229 (11)
C13	0.0884 (18)	0.121 (2)	0.106 (2)	−0.0729 (18)	−0.0038 (15)	−0.0332 (17)
C14	0.097 (2)	0.0794 (18)	0.154 (3)	−0.0341 (16)	−0.0215 (19)	−0.0580 (18)
C15	0.119 (2)	0.130 (2)	0.100 (2)	−0.064 (2)	−0.0581 (19)	−0.0060 (18)

### Geometric parameters (Å, °)

**Table:**

C1—C2	1.360 (3)	C9—H9A	0.96
C1—C6	1.368 (3)	C10—H10A	0.96
C1—H1	0.93	C10—H10B	0.96
C2—C3	1.387 (3)	C10—H10C	0.96
C2—H2	0.93	C11—O2	1.197 (2)
C3—C4	1.391 (3)	C11—O3	1.336 (2)
C3—H3	0.93	O3—C12	1.479 (2)
C4—C5	1.381 (3)	C12—C15	1.504 (3)
C4—C7	1.505 (3)	C12—C14	1.507 (3)
C5—C6	1.382 (3)	C12—C13	1.514 (3)
C5—H5	0.93	C13—H13A	0.96
C6—H6	0.93	C13—H13B	0.96
C7—O1	1.212 (2)	C13—H13C	0.96
C7—C8	1.535 (3)	C14—H14A	0.96
C8—C11	1.522 (3)	C14—H14B	0.96
C8—C10	1.540 (3)	C14—H14C	0.96
C8—C9	1.540 (3)	C15—H15A	0.96
C9—H9B	0.96	C15—H15B	0.96
C9—H9C	0.96	C15—H15C	0.96
			
C2—C1—C6	120.1 (2)	C8—C10—H10B	109.5
C2—C1—H1	119.9	H10A—C10—H10B	109.5
C6—C1—H1	119.9	C8—C10—H10C	109.5
C1—C2—C3	120.2 (2)	H10A—C10—H10C	109.5
C1—C2—H2	119.9	H10B—C10—H10C	109.5
C3—C2—H2	119.9	O2—C11—O3	125.43 (18)
C2—C3—C4	120.3 (2)	O2—C11—C8	125.07 (17)
C2—C3—H3	119.8	O3—C11—C8	109.46 (16)
C4—C3—H3	119.8	C11—O3—C12	122.50 (15)
C5—C4—C3	118.4 (2)	O3—C12—C15	109.11 (18)
C5—C4—C7	123.82 (18)	O3—C12—C14	109.65 (17)
C3—C4—C7	117.79 (18)	C15—C12—C14	113.7 (2)
C4—C5—C6	120.6 (2)	O3—C12—C13	102.51 (16)
C4—C5—H5	119.7	C15—C12—C13	110.8 (2)
C6—C5—H5	119.7	C14—C12—C13	110.4 (2)
C1—C6—C5	120.3 (2)	C12—C13—H13A	109.5
C1—C6—H6	119.8	C12—C13—H13B	109.5
C5—C6—H6	119.8	H13A—C13—H13B	109.5
O1—C7—C4	119.24 (19)	C12—C13—H13C	109.5
O1—C7—C8	119.62 (19)	H13A—C13—H13C	109.5
C4—C7—C8	121.14 (16)	H13B—C13—H13C	109.5
C11—C8—C7	110.39 (15)	C12—C14—H14A	109.5
C11—C8—C10	111.49 (17)	C12—C14—H14B	109.5
C7—C8—C10	109.80 (16)	H14A—C14—H14B	109.5
C11—C8—C9	106.87 (16)	C12—C14—H14C	109.5
C7—C8—C9	109.79 (17)	H14A—C14—H14C	109.5
C10—C8—C9	108.42 (17)	H14B—C14—H14C	109.5
C8—C9—H9B	109.5	C12—C15—H15A	109.5
C8—C9—H9C	109.5	C12—C15—H15B	109.5
H9B—C9—H9C	109.5	H15A—C15—H15B	109.5
C8—C9—H9A	109.5	C12—C15—H15C	109.5
H9B—C9—H9A	109.5	H15A—C15—H15C	109.5
H9C—C9—H9A	109.5	H15B—C15—H15C	109.5
C8—C10—H10A	109.5		

### Hydrogen-bond geometry (Å, °)

**Table:**

*D*—H···*A*	*D*—H	H···*A*	*D*···*A*	*D*—H···*A*
C5—H5···O2^i^	0.93	2.66	3.317 (3)	128
C9—H9A···O1^ii^	0.96	2.65	3.557 (3)	158

Symmetry codes: (i) −*x*+1, −*y*, −*z*+2; (ii) −*x*+1, −*y*, −*z*+1.
